# Support for Expanding Access to Cannabis Among Physicians and Adults With Chronic Pain

**DOI:** 10.1001/jamanetworkopen.2024.35843

**Published:** 2024-09-26

**Authors:** Elizabeth M. Stone, Kayla Tormohlen, Mark C. Bicket, Emma E. McGinty

**Affiliations:** 1 Center for Health Services Research, Rutgers Institute for Health, Health Care Policy, and Aging Research, New Brunswick, New Jersey; 2 Department of Psychiatry, Rutgers Robert Wood Johnson Medical School, New Brunswick, New Jersey; 3 Division of Healthcare Policy and Economics, Weill Cornell Medical College, New York, New York; 4 Department of Anesthesiology, University of Michigan, Ann Arbor; 5 Opioid Prescribing Engagement Network, Institute for Healthcare Policy and Innovation, University of Michigan School of Public Health, University of Michigan, Ann Arbor

## Abstract

This survey study reports opinions of patients with chronic pain and physicians who treat chronic pain on policies regarding access to cannabis for chronic pain management.

## Introduction

Most states have legalized medical cannabis; many have legalized cannabis for adult recreational use.^1^ Given this policy environment, we conducted surveys of physicians and people with chronic pain on support for policies affecting access to cannabis for chronic pain.

## Methods

We conducted surveys on cannabis for chronic pain among adults with chronic pain and physicians who treated chronic pain in states with medical cannabis programs at the time of the first survey (eTable 1 in Supplement 1). People with chronic pain were surveyed from March 3 to April 11, 2022, using the NORC AmeriSpeak panel.^2^ Physicians were surveyed from July 13 to August 4, 2023, by Ipsos using the Survey Healthcare Global physician survey panel.^3^ Response rates were 75.4% for people with chronic pain and 73.0% for physicians (eMethods in Supplement 1). This study was approved by the Weill Cornell Medical College institutional review board and followed the AAPOR reporting guideline and Best Practices for Survey Research by reporting all Transparency Initiative Disclosure Elements. For both groups, completion of the survey indicated consent to participate.

Respondents were asked if they favored or opposed policies expanding access to and increasing regulations on cannabis (eTable 2 in Supplement 1). Responses were measured on a 5-point Likert scale, with responses of “strongly favor” or “favor” indicating support. We compared policy support between people with chronic pain and physicians and by prior use or recommendation of cannabis for chronic pain management.

Differences in policy support were assessed using χ^2^ tests. All analyses used survey weights to generate estimates representative of the general adult and physician populations. Analyses were conducted using Stata, version 18.^4^ All *P* values were from 2-sided tests and deemed statistically significant at *P* < .05.

## Results

Respondents included 1661 people with chronic pain (mean [SD] age, 52.3 [16.9] years; 53.4% women and 46.6% men; 3.0% Asian, 11.3% Black, 16.8% Hispanic or Latino, 66.2% White, and 2.8% race or ethnicity other than those listed) and 1000 physicians (mean [SD] age, 51.9 [11.3] years; 63.0% women, 34.5% men, 0.7% nonbinary, transgender, genderqueer, or gender fluid, and 1.9% prefer not to say; 23.1% Asian, 6.3% Black, 6.5% Hispanic or Latino, 63.2% White, and 4.7% other race or ethnicity). Compared with physicians, more people with chronic pain supported 4 policies expanding access to cannabis: federal legalization of medical cannabis (70.8% vs 59.0%; *P* < .001), federal legalization of cannabis for adult use (54.9% vs 38.0%; *P* < .001), requiring insurance coverage of cannabis for chronic pain treatment (64.0% vs 50.6%; *P* < .001), and requiring states with medical cannabis programs to provide subsidies for people with low income (50.1% vs 30.6%; *P* < .001) (Figure). Fewer people with chronic pain supported requiring patient registration with the state medical cannabis program to access medical cannabis (49.2% vs 68.1%; *P* < .001).

**Figure.**
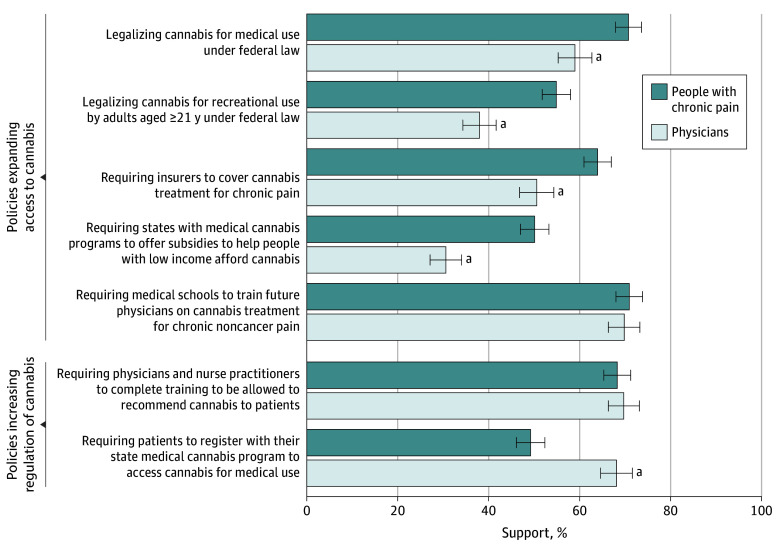
Support for Cannabis Policies Among Physicians and People With Chronic Pain Error bars indicate 95% CIs. ^a^*P* < .001.

People who used cannabis for chronic pain reported the highest levels of support for policies expanding access; physicians who had not recommended cannabis for chronic pain reported the lowest levels (Table). More physicians who had (vs had not) recommended cannabis for people with chronic pain supported the 2 measures increasing regulation of medical cannabis (physician training: 74.3% vs 63.2%; *P* = .002; patient registration with state medical cannabis programs: 71.2% vs 63.7%; *P* = .04). Fewer people who used cannabis for chronic pain (vs not) supported patient registration requirements (41.0% vs 53.5%; *P* < .001).

**Table.** Support for Cannabis Policies

Policy	People with chronic pain, ever used cannabis			Physicians, past-year recommended cannabis		
	Yes (n = 495), % (95% CI)	No (n = 1166), % (95% CI)	*P* value	Yes (n = 618), % (95% CI)	No (n = 382), % (95% CI)	*P* value
Legalizing cannabis for medical use under federal law	89.4 (85.9-92.8)	63.2 (59.5-66.8)	<.001	71.7 (67.2-76.1)	40.9 (34.9-46.9)	<.001
Legalizing cannabis for recreational use by adults aged ≥21 y under federal law	79.1 (74.4-83.7)	44.2 (40.5-47.9)	<.001	47.4 (42.6-52.9)	24.6 (19.2-29.9)	<.001
Requiring insurers to cover cannabis treatment for chronic pain	80.1 (75.4-84.8)	57.4 (53.7-61.1)	<.001	63.2 (58.5-67.8)	32.6 (26.8-38.4)	<.001
Requiring states with medical cannabis programs to offer subsidies to help people with low income afford cannabis	64.6 (59.1-70.2)	44.0 (40.3-47.7)	<.001	41.1 (36.7-45.9)	15.6 (11.1-20.1)	<.001
Requiring medical schools to train future physicians on cannabis treatment for chronic noncancer pain	76.9 (71.5-82.2)	69.0 (65.5-72.5)	.02	79.2 (75.2-83.2)	56.4 (50.5-62.3)	<.001
Requiring physicians and nurse practitioners to complete training to be allowed to recommend cannabis to patients	72.7 (67.3-78.1)	66.9 (63.3-70.5)	.77	74.3 (70.2-78.5)	63.2 (57.4-68.9)	.002
Requiring patients to register with their state medical cannabis program to access cannabis for medical use	41.0 (35.2-46.8)	53.5 (49.8-57.2)	<.001	71.2 (66.9-75.5)	63.7 (58.0-69.4)	.04

## Discussion

Compared with physicians, adults with chronic pain reported greater support for policies expanding access to cannabis and less support for policies further regulating medical cannabis. People who used cannabis for chronic pain were most in favor of expanding access, while physicians who had not recommended cannabis to people with chronic pain reported the least support. Most respondents supported training requirements for medical students and physicians on the use of cannabis for chronic noncancer pain.^5,6^ Limitations of this study include the possibility of sampling bias in the web panels used and self-report biases related to issues with recall or social desirability.

## Notes

Supplement 1. **eTable 1.** List of States and Districts With Active Medical Cannabis Programs as of March 2022 **eMethods.** Detailed Description of Survey Methods **eTable 2.** Survey Questions

Supplement 2.
Data Sharing Statement
