# Supplementary material for: Support for Expanding Access to Cannabis Among Physicians and Adults With Chronic Pain
Source: JAMA Netw Open. 2024 Sep 26;7(9):e2435843. doi: 10.1001/jamanetworkopen.2024.35843 (PMC11427956; doi:10.1001/jamanetworkopen.2024.35843)
Supplement: Supplement 1. — eTable 1. List of States and Districts With Active Medical Cannabis Programs as of March 2022 eMethods. Detailed Description of Survey Methods eTable 2. Survey Questions [file jamanetwopen-e2435843-s001.pdf]

Supplementary Online Content

Stone EM, Tormohlen K, Bicket MC, McGinty EE. Support for expanding access to cannabis among physicians and adults with chronic pain. *JAMA Netw Open.* 2024;7(9):e2435843. doi:10.1001/jamanetworkopen.2024.35843

eTable 1. List of States and Districts With Active Medical Cannabis Programs as of March 2022

eMethods. Detailed Description of Survey Methods

eTable 2. Survey Questions

This supplementary material has been provided by the authors to give readers additional information about their work.

eTable 1. List of States and Districts With Active Medical Cannabis Programs as of March 2022

Alaska
Alabama
Arkansas
Arizona
California
Colorado
Connecticut
District of Columbia
Delaware
Florida
Hawaii
Illinois
Louisiana
Massachusetts
Maryland
Maine
Michigan
Minnesota
Missouri
Montana
North Dakota
New Hampshire
New Jersey
New Mexico
Nevada
New York
Ohio
Oklahoma
Oregon
Pennsylvania
Rhode Island
South Dakota
Utah
Virginia
Vermont
Washington
West Virginia

© 2024 Stone EM et al. *JAMA Network Open.*

eMethods. Detailed Description of Survey Methods

Survey of People with Chronic Pain

This was survey was developed, reviewed, and refined by a study team including survey methods experts, policy experts, and pain clinicians. Policy support items included in this survey were selected based on a review of the literature by study team members. The survey was fielded using the NORC AmeriSpeak panel. This is a probability-based panel covering 97% of U.S. households with a 34% weighted household response rate for panel recruitment. The current panel includes 54,001 members aged 13 and over. Once enrolled in the panel, members are invited to participate in web- or phone-based studies 2-3 times per month. For our study, individuals were eligible to participate if 1) they lived in one of the 36 states and Washington DC with active medical cannabis programs at the time of the survey and 2) they had chronic noncancer pain. We defined chronic noncancer pain using the National Health Interview Survey criterion of having pain unrelated to cancer on most days or every day in the past 6 months. A pilot test of 100 panelists was conducted on February 21, 2022. The study team reviewed pilot responses for quality control (e.g., appropriate skip patterns). Pilot responses were not included in the final analysis. The main survey was fielded from March 3 to April 11, 2022. Eligibility for participation was assessed in a screener survey with a response rate of 75%. Of the 1724 people determined to be eligible for participation based on the screener survey, 1661 (96.3%) completed the survey.

Survey of Physicians

This was survey was developed, reviewed, and refined by a study team including survey methods experts, policy experts, and pain clinicians. Policy support items included in this survey were selected based on a review of the literature by study team members. The survey was fielded using the Ipsos Survey Healthcare Global physician survey panel. The is an opt-in panel including approximately 800,000 U.S. physicians recruited from the American Medical Association (AMA) membership list and hospital and other verified medical directories of physicians. For our study, physicians were eligible to participate if they 1) were in a specialty that commonly treats chronic noncancer pain (family medicine, internal medicine, general medicine, anesthesiology, neurology, physical medicine, and rehabilitation), 2) reported spending 50% or more of their professional time care for patients, 3) reported caring for 100 or more patients in the past year, and 4) reported caring for any patients with chronic noncancer pain in an outpatient clinical setting in the past year. The main survey was fielded from July 13 to August 4, 2023. The first 30 respondents constituted a “soft launch” period. Following this period, reviews were conducted for quality control (e.g., questionnaire logic, length of interview) prior to full survey launch. A screening survey identified eligible physicians who were then invited via email to participate in the survey. The response rate for this survey was 73%.

© 2024 Stone EM et al. *JAMA Network Open.*

eTable 2. Survey Questions

Question	Response options	Survey of People with Chronic Pain	Survey of Physicians
**Screening Questions**			
In the past 6 months, how often did you have pain? Would you say never, some days, most days, or every day?	Never<br>Some days<br>Most days<br>Every day<br>DON'T KNOW	X	
Is your pain related to cancer?	Yes<br>No<br>DON'T KNOW	X	
Do you still reside in [INSERT [STATE]]?	Yes<br>No<br>DON'T KNOW	X	
If no, what state do you reside in?	DON'T KNOW<br>Alaska<br>Alabama<br>Arkansas<br>Arizona<br>California<br>Colorado<br>Connecticut<br>District of Columbia<br>Delaware<br>Florida<br>Georgia<br>Hawaii<br>Iowa<br>Idaho<br>Illinois<br>Indiana<br>Kansas<br>Kentucky<br>Louisiana<br>Massachusetts<br>Maryland<br>Maine<br>Michigan<br>Minnesota<br>Missouri<br>Mississippi<br>Montana<br>North Carolina<br>North Dakota<br>Nebraska<br>New Hampshire<br>New Jersey<br>New Mexico<br>Nevada<br>New York<br>Oregon<br>Ohio<br>Oklahoma	X	

© 2024 Stone EM et al. *JAMA Network Open.*

	Pennsylvania<br>Rhode Island<br>South Carolina<br>South Dakota<br>Tennessee<br>Texas<br>Utah<br>Virginia<br>Vermont<br>Washington<br>Wisconsin<br>West Virginia<br>Wyoming		
Would you be willing to complete this survey?	I agree to participate<br>I do not agree to participate<br>DON'T KNOW	X	
In the past year, what proportion of your clinical time has involved the management of acute or chronic conditions for a panel of patients?	Less than 50%<br>50% or more		X
In the past year, have you evaluated or treated at least 100 patients?	Yes<br>No		X
In the past year, have you evaluated or treated any patients with chronic noncancer pain in an office or other outpatient clinical setting?	Yes<br>No		X
In the past year, have you recommended medical cannabis for your patients' noncancer chronic pain?	Yes<br>No		X
**Cannabis Questions**			
Have you ever used cannabis to manage your chronic pain?	Yes<br>No<br>DON'T KNOW	X	
In the past year, have you recommended medical cannabis for your patients' noncancer chronic pain?	Yes<br>No		X
**Policy Support**			
Do you favor or oppose legalizing cannabis for medical use under federal law?	Strongly favor<br>Favor<br>Neither favor nor oppose<br>Oppose<br>Strongly oppose	X	X
Do you favor or oppose legalizing cannabis for recreational use by adults aged 21 years or older under federal law?	Strongly favor<br>Favor<br>Neither favor nor oppose<br>Oppose<br>Strongly oppose	X	X
Do you favor or oppose requiring insurers to cover cannabis treatment for chronic pain?	Strongly favor<br>Favor<br>Neither favor nor oppose<br>Oppose<br>Strongly oppose	X	X
Do you favor or oppose requiring states with medical cannabis programs	Strongly favor<br>Favor<br>Neither favor nor oppose	X	X

© 2024 Stone EM et al. *JAMA Network Open.*

to offer subsidies to help low-income people afford cannabis?	Oppose<br>Strongly oppose		
Do you favor or oppose requiring physicians to receive training on use of cannabis for chronic pain?	Strongly favor<br>Favor<br>Neither favor nor oppose<br>Oppose<br>Strongly oppose	X	X
Do you favor or oppose requiring physicians and nurse practitioners to complete training in order to be allowed to recommend cannabis to patients?	Strongly favor<br>Favor<br>Neither favor nor oppose<br>Oppose<br>Strongly oppose	X	X
Do you favor or oppose requiring patients to register with their state medical cannabis program to access cannabis for medical use?	Strongly favor<br>Favor<br>Neither favor nor oppose<br>Oppose<br>Strongly oppose	X	X

© 2024 Stone EM et al. *JAMA Network Open.*
