# Supplementary material for: Support for Expanding Access to Cannabis Among Physicians and Adults With Chronic Pain
Source: JAMA Netw Open. 2024 Sep 26;7(9):e2435843. doi: 10.1001/jamanetworkopen.2024.35843 (PMC11427956; doi:10.1001/jamanetworkopen.2024.35843)
Supplement: Supplement 2. — Data Sharing Statement [file jamanetwopen-e2435843-s002.pdf]

Data Sharing Statement

Stone. Support for Expanding Access to Cannabis Among Physicians and Adults With Chronic Pain. *JAMA Netw Open*. Published September 26, 2024.
doi:10.1001/jamanetworkopen.2024.35843

Data
Data available: No

Additional Information
Explanation for why data not available: The data will not be shared per the data use agreements with NORC and Ipsos, which only allow access by the study team.
